# Thrombin-Accelerated Quick Clotting Serum Tubes: An Evaluation with 22 Common Biochemical Analytes

**DOI:** 10.1155/2013/769479

**Published:** 2013-04-03

**Authors:** Wai-Yoong Ng, Chin-Pin Yeo

**Affiliations:** Department of Pathology, Clinical Biochemistry Laboratories, Singapore General Hospital, Outram Road, Singapore 169608

## Abstract

Clot activator serum tubes have significantly improved turnaround times for result reporting compared to plain tubes. With increasing workload and service performance expectations confronting clinical laboratories with high-volume testing and with particular emphasis on critical analytes, attention has focussed on preanalytical variables that can be improved. We carried out a field study on the test performance of BD vacutainer rapid serum tubes (RSTs) compared to current institutional issued BD vacutainer serum separator tubes (SSTs) in its test result comparability, clotting time, and stability on serum storage. Data from the study population (*n* = 160) of patients attending outpatient clinics and healthy subjects showed that results for renal, liver, lipids, cardiac, thyroid, and prostate biochemical markers were comparable between RSTs and SSTs. Clotting times of the RSTs were verified to be quick with a median time of 2.05 min. Analyte stability on serum storage at 4°C showed no statistically significant deterioration except for bicarbonate, electrolytes, and albumin over a period of 4 days. In conclusion, RSTs offered savings in the time required for the clotting process of serum specimens. This should translate to further trimming of the whole process from blood collection to result reporting without too much sacrifice on test accuracy and performance compared to the current widely used SSTs in most clinical laboratories.

## 1. Introduction

The performance of laboratory services is often expected to demonstrate delivery of accurate test results in the shortest possible time. To meet service targets, turnaround times are constantly under scrutiny for further improvement. What is perhaps less understood are the various influences that determine the prompt delivery of results from the journey of the blood specimen which begins at the blood collection point and continues to testing and result reporting. Studies have indicated that much of the delay is due to factors at the preanalytical phase including specimen delivery efficiencies [1, 2]. A key factor is the clotting time of blood collected for serum testing, and current blood collection tubes (with clot activator and gel separation) require a recommended 30-min standing time for clotting. Hence, at times, specimens reaching the laboratory through a fast delivery service (example, for STAT specimens) may not have clotted completely to allow immediate testing.

To mitigate the clotting time issue, some laboratories are using plasma specimens for STAT testing. However, it is known that plasma is not exactly an adequate substitute for serum [3, 4]. Plasma has its own set of areas for concern, for example, problem of adequate mixing of blood with anticoagulants, difference in result values for some analytes when measured in serum or plasma, especially potassium and lactate dehydrogenase, thus requiring changes in reference intervals [5]. With its inherent known limitations particularly in the more recent reports of issues with cardiac markers, plasma may not be the specimen of choice [6–9].

The Becton Dickinson BD vacutainer rapid serum tube (RST BD 368774) has a 5-min clotting time which is advantageous for any immediate processing in the high-volume clinical laboratory. Commercially available in 2010 with supporting data on its use on various popular instrument platforms, more recent studies have shown that RSTs can replace plasma gel tubes [10]. The RSTs were shown to produce equivalent Troponin-T results compared to SSTs [11, 12] and give less false-positive Troponin-I and Troponin-T results compared to plasma gel tubes [13, 14]. Manufacturer's white papers have supporting data that RSTs showed same results as current serum tubes or plasma tubes except for some analytes, for example, total protein, hence, a suitable alternative to the use of plasma gel tubes in the STAT environment.

As RSTs were recently introduced in our region, we carried out a field study on its quick clotting performance and tests accuracies of routine chemistries often requested by the emergency department and outpatient clinics. The RSTs were compared with the current SSTs (serum separation tube BD 367986) on test results for general biochemical indices such as renal, liver, lipids, cardiac markers, and thyroid function. A subset of archived samples (at 4°C) was further evaluated for analyte stability by retesting for the same analytes each day for up to 5 days.

## 2. Methods and Materials

Blood specimens were collected from adult healthy subjects (*n* = 85) and patients (*n* = 75) for routine chemistries—renal panel, liver panel, lipid panel, cardiac markers, thyroid hormones, and prostate specific antigen. Patients were randomly selected at the outpatient clinics for participation. Both blood specimens in RSTs and current institutional issued SSTs were obtained from the participants in no particular order with only the order of draw (for various specimen type) adhered to for those patients with various other tests requested. Blood tubes were mixed with 5 inversions as a standard protocol before dispatched to the clinical laboratories for testing.

To determine the clotting time, an empirical visual approach was taken to manually time the clotting process for some RST and SST (*n* = 30). Tube pairs (RST, SST) were placed side by side with occasional tilting of the tubes to check on the clot process. Once a firm clot (>50% no flow) was shown, the timer (on a stop watch) was stopped.

### 2.1. Workflow and Analyte Testing

Following accessioning of requested tests, the workflow for patient samples followed the usual process through the central lab automation system where specimen tubes are placed through the automated routing process of centrifugation, aliquoting, testing in linked analyzers, and finally archived in the specimen stockyard. For healthy subject specimens, they were manually processed for centrifugation and testing.

RST and SST specimen tubes for patients processed through the central lab automation system were analyzed on the automation-line-connected Beckman Coulter DxC 800 analyzers for the renal panel (urea, creatinine, bicarbonate, sodium, potassium, chloride, and glucose), liver panel (total protein, albumin, total bilirubin, alkaline phosphatase ALP, alanine aminotransferase ALT, and aspartate aminotransferase AST), and lipid panel (total cholesterol, HDL-cholesterol, triglycerides) and DxI 800 for thyroid hormones (thyroid stimulating hormone TSH, free thyroxine fT4). Sera from healthy subjects for the same analytes (renal, liver, and lipids) were analyzed on the Roche MODULAR EVO analyzer. Thyroid hormones for both subject populations were analyzed on the DxI 800. The cardiac markers (Troponin-T, creatine kinase-MB CK-MB, and N-terminal brain natriuretic peptide NT-proBNP) and prostate-specific antigen (PSA) were analyzed on a Roche cobas6000 analyzer.

### 2.2. Stability Study

Paired blood specimens collected in RSTs and SSTs (*n* = 31) that were tested for renal, liver, and lipid panels, cardiac markers and thyroid hormones were archived at 4°C (D1). The samples were re-tested for the same analytes each day (D2–D5) for the next 4 days. Storage of the study samples was in the same cold room as other routine blood specimens.

### 2.3. Data Analysis

Results were analyzed using Passing-Bablok and Bland-Altman plots for association and differences. Tube comparison results analyzed by the Student's *t*-test were applied for each analyte. Analysis of variance (ANOVA) procedure for group mean differences against the initial first day D1 over the next 4 days' (D2–D5) results for serum stability at different days of storage was performed. Differences are considered statistically significant at a *P* value of <0.05.

 This study has institutional ethics approval (CIRB2011/855/B) with informed consent from all participants.

## 3. Results and Discussion

All study analytes from the healthy subjects were readily tested; however, not all of these analytes were requested for the outpatients, hence, the unequal number of tests for each analyte (85–129 results). Subjects' demographics gave the following: 70 males, 90 females of average age 43 years (range 20–77). There were 85 healthy subjects and 75 patients. A summary of the tests and instrument platform associated is shown in Table 1.

Visual review of the clotting process clearly verified a much shorter clot formation in the RSTs at less than 5 min compared to SSTs. Of the 30 RSTs and SSTs observed, clots formed almost immediately for the RSTs. The RSTs gave an overall mean clotting time of 2.07 min (range 1–4 min, median 2.05 min). Blood in SSTs did not clot at 5 min and most required at least 15 min to show signs of clotting.

Most parallel test results for RSTs and SSTs agreed very well. Means of the RSTs were in close agreement with that of the control (SST) tube for all 22 analytes (Table 2). No statistical significant differences in results were indicated for the RSTs compared to SSTs. It is noted that absolute differences were small, for example, potassium −0.5 to +0.5 mmol/L although translated in percent difference would be large. As shown in the table, results for renal and liver panels that are universally requested were similarly observed on both types of blood collection tubes. Results for cardiac markers were also of similar mean values between the two tubes.

**Figure 1:**
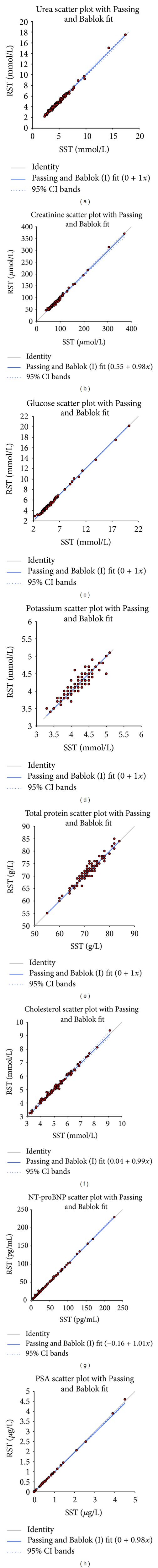
Comparison of results for RST and SST for the select analytes. Select analytes: Passing-Bablok regression analysis. (a) urea, (b) creatinine, (c) glucose, (d) potassium, (e) total protein, (f) cholesterol, (g) NT-proBNP, and (h) PSA.

**Figure 2:**
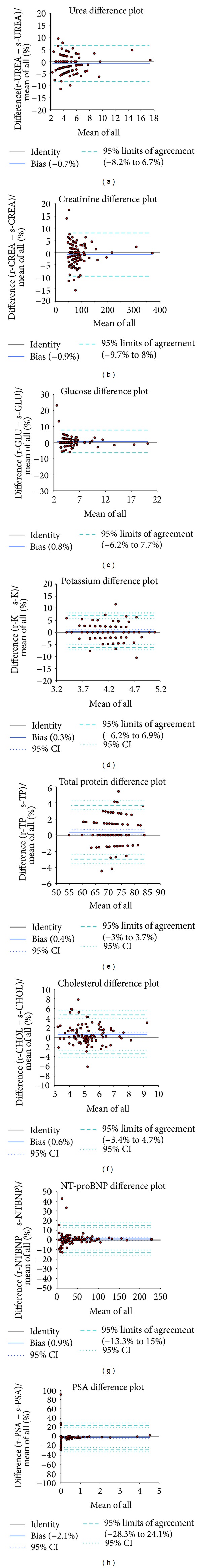
Bland-Altman difference plots for select analytes. Select analytes: (a) urea, (b) creatinine, (c) glucose, (d) potassium, (e) total protein, (f) cholesterol, (g) NT-proBNP, and (h) PSA.

Graphical illustrations of the select analytes chosen for their high workload, requirement of short turnaround time, and panic value reporting especially in the accident and emergency setting (Figure 1), showed strong correlations between RSTs and SSTs as evidenced by near equivalent slopes as determined with Passing-Bablok regression fits. Most of the tests gave a slope of 1.00 and zero *y*-intercepts as summarised in Table 3. Selected analytes also displayed these tight fittings with Spearman's correlations ranging from 0.91 to 1.00. Bland-Altman difference plots showed low mean biases in general (Figure 2, Table 3). In the case for Troponin-T, none of the results were reported as positive (>0.03 *μ*g/L), and the few results (*n* = 8) that were >0.003 *μ*g/L (lowest detectable level) still gave a Passing-Bablok slope of 1.00.

Stability studies of archived specimens showed that serum in RSTs has the same stability as in SSTs at 4°C, tested up to 5 days for the renal, liver, and lipid panels, cardiac markers, and thyroid hormones except for bicarbonate whose levels differ substantially each day and would have clinical impact (Table 4). The mean values for bicarbonate decrease daily with large differences between days with statistical significance shown immediately on the next day D2, averaging −25.6% to 7.2% and corresponding medians of −25.8% to 1.1%. For the electrolytes sodium, potassium, and chloride, differences between days were less than 12%, averaging 0.7% to 6.2% with corresponding median values of 0% to 6.8%. Though statistically significant at D3 and D4, the differences have little clinical impact. This is also shown with albumin, another analyte that showed some incremental increase on storage but of little clinical impact.

Although insufficient serum was available to test for all analytes on the fifth day of storage (D5; only 8 cases have results), similar group means were obtained and included in the ANOVA computation (data not shown). The result trending of the analytes was similar to D2–D4, that is, all ANOVA tested analytes showed no statistical significant difference except for bicarbonate, sodium, potassium, chloride, and albumin.

Turnaround times for test results depend on how fast the test can be performed, how fast the blood sample reaches the laboratory, how fast the laboratory processes the specimen, and how fast the results are reported. Hence, there are potential areas for improvement at the preanalytical, analytical, and postanalytical phases. There have been various studies on the preanalytical phase with blood collection tubes being one of the improvement areas [1, 2, 15]. For a fast turnaround for tests from the emergency department and outpatient clinics, use of plasma tubes is one answer to the limitation of a 30-minute clotting time imposed by the standard blood collection tubes with serum separator and clot activator.

In addition, RSTs have also been shown to give comparable results for reproductive hormones (e.g., follicle stimulating hormone, luteinizing hormone, human chorionic gonadotropin, and estradiol) and similarly for cardiac markers and thyroid hormones, on other instrument platforms—Siemens ADVIA Centaur, Beckman Access II, Abbott AxSYM, and Ortho Clinical Vitros eCi (data not shown).

It should, however, be clearly made known that as thrombin is used to accelerate clotting, the endogenous pathways involving thrombin would be affected with these tubes. Those patients for coagulation studies are likely to be affected and should not have their blood collected with the RSTs. Similarly, RSTs are not recommended for patients on heparin therapy, thrombin inhibitor therapy, or with deficiency in the clotting factors as latent clotting has been observed on patients on high dose heparin treatment resulting with APTT > 150 sec [10]. In our study, none of the 75 outpatients recruited for this study had coordered coagulation tests on the day of study participation that would suggest any recent high dose heparin therapy typically administered in an inpatient setting. It would, however, be prudent to confine the use of RSTs which are primarily suited for fast turnaround needs, to subjects without recent history of coagulation treatment, for example, cardiac surgery.

## 4. Conclusions

This present study has observed concordance of common analytes on evaluation of serum from the standard blood collection tubes and the quick clotting tubes. The new RSTs have also been reported to give very similar results as SSTs [10, 16]. With quick clotting, RSTs potentially allow time gains in terms of reduced turnaround times and circumvent the use of plasma specimens to achieve service performance improvements. As observed in other studies, the rapid clotting tubes, RSTs, could bridge the concern of short turnaround service performance for many healthcare institutions that have converted to the use of plasma instead of serum.

A notable aspect of our study depicts hospital-based clinical practice conditions for the collection of blood specimens and real situation tests' performance. In summary, RSTs gave comparable test results as current serum separator tubes for some of the most common biochemical analytes ordered in the emergency and outpatient setting. This tube development would be helpful to achieve short turnaround times for serum specimens needing STAT reporting.

## Figures and Tables

**Table 1:** Tests associated with instrument platforms.

	Group	Principle	Instruments for health, patients
Urea, creatinine	Renal	Chemistry	MOD, DxC800
Bicarbonate, glucose			
Sodium, potassium, chloride	Electrolyte	Electrochemistry	MOD, DxC800
Total protein, albumin	Liver	Chemistry	MOD, DxC800
Total Bilirubin, ALP, ALT, AST			
Cholesterol, HDL, triglycerides	Lipid	Chemistry	MOD, DxC800
TSH, free T4	Thyroid	Immunoassay	DxI800
Troponin T, CK-MB, NT-proBNP	Cardiac	Immunoassay	cobas6000
PSA	Prostate	Immunoassay	cobas6000

Beckman Coulter: DxC800, DxI800.

Roche Diagnostics: MODULAR EVO, cobas6000.

**Table 2:** Summary statistics for RST results compared with control (SST) tubes.

Analyte	Units	*n*	RST mean	Range	SST mean	Range	Diff mean	95% CI range	*t*-test *P* value
Urea	mmol/L	106	4.8	2.2, 17.5	4.9	2.3, 17.4	0.0	−0.6, 0.7	0.90
Creatinine	*μ*mol/L	127	81.9	46, 370	82.7	40, 371	−0.7	−12, 9	0.89
Bicarbonate	mmol/L	104	24.9	15.4, 29.9	25.0	19.0, 29.9	−0.1	−5.8, 3.8	0.71
Glucose	mmol/L	97	5.4	2.9, 20.2	5.4	2.3, 20.3	0.0	−0.3, 0.6	0.94
Sodium	mmol/L	106	140.1	132, 146	140.3	133, 148	−0.2	−4, 4	0.52
Potassium	mmol/L	108	4.2	3.4, 5.1	4.2	3.3, 5.1	0.0	−0.5, 0.5	0.70
Chloride	mmol/L	106	103.7	99, 110	103.7	98, 109	0.0	−4, 3	1.00
Total protein	g/L	107	71.9	55, 85	71.6	55, 84	0.3	−3, 4	0.69
Albumin	g/L	127	39.1	24, 49	38.8	24, 48	0.3	−2, 2	0.52
Total Bilirubin	*μ*mol/L	110	11.2	4.0, 33.0	11.4	4.4, 34.0	−0.2	−19, 4	0.80
ALP	U/L	108	66.3	28, 338	66.4	35, 331	−0.2	−13, 7	0.97
ALT	U/L	129	25.7	4, 374	25.6	4, 375	−0.2	−5, 7	0.95
AST	U/L	121	25.3	8, 465	25.6	10, 451	−0.3	−5, 14	0.96
Cholesterol	mmol/L	93	5.21	3.23, 9.39	5.18	3.17, 9.11	0.03	−0.32, 0.36	0.84
HDL	mmol/L	93	1.47	0.10, 2.72	1.48	0.66, 2.75	−0.01	−0.95, 0.20	0.86
Triglycerides	mmol/L	93	1.18	0.41, 4.83	1.16	0.41, 4.70	0.02	−0.12, 0.13	0.88
TSH	mU/L	98	2.125	0.015, 43.4	2.040	0.015, 39.9	0.085	−0.406, 3.50	0.88
fT4	pmol/L	98	11.7	4.0, 19.5	11.7	4.4, 19.4	−0.04	−3.3, 2.4	0.88
Troponin-T	*μ*g/L	86	0.003	0.003, 0.01	0.003	0.003, 0.01	−0.0002	−0.013, 0	0.44
CK-MB	*μ*g/L	86	1.86	0.71, 5.65	1.89	0.78, 5.71	−0.03	−0.19, 0.24	0.85
NT-proBNP	pg/mL	85	44.4	5.0, 229	44.1	5.0, 228	0.33	−2.6, 7.71	0.95
PSA	*μ*g/L	87	0.33	0.003, 4.60	0.34	0.003, 4.50	0.00	−0.06, 0.10	0.97

Diff: absolute difference (RST − SST).

95% CI: minimum, maximum.

Range: minimum, maximum.

**Table 3:** Summary of Passing-Bablok regression and Bland-Altman bias between RST and SST.

Analyte		Passing-Bablok		Correl		95% CI bias		95% limitAgrmt	
	Sample size	Slope	Constant	rs	mBias%	Min.	Max.	Min.	Max.
Urea	106	1.00	0.00	0.99	−0.7	−1.5	−0.0	−8.2	6.7
Creatinine	127	0.98	0.55	0.99	−0.9	−1.7	−0.1	−9.7	8.0
Bicarbonate	104	1.00	0.00	0.86	−0.5	−1.6	0.5	−11.1	10.0
Glucose	97	1.00	0.00	0.99	0.8	0.1	1.5	−6.2	7.7
Sodium	106	1.00	0.00	0.82	−0.2	−0.4	0.0	−2.2	1.8
Potassium	108	1.00	0.00	0.91	0.3	−0.3	1.0	−6.2	6.9
Chloride	106	1.00	0.00	0.83	0.0	−0.2	0.2	−2.4	2.4
Total protein	107	1.00	0.00	0.95	0.4	0.0	0.7	−3.0	3.7
Albumin	127	1.00	0.00	0.98	0.7	0.4	1.0	−2.7	4.0
Total Bilirubin	110	1.00	0.00	0.94	−1.5	−4.4	1.3	−31.4	28.4
ALP	108	1.00	0.00	0.99	−0.6	−1.4	0.2	−8.9	7.8
ALT	129	1.00	0.00	0.98	−2.1	−3.9	−0.3	−22.3	18.2
AST	121	1.00	0.00	0.96					
Cholesterol	93	0.99	0.04	0.99	0.6	0.2	1.1	−3.4	4.7
HDL	93	1.02	−0.03	0.99	−2.0	−5.6	1.6	−36.3	32.2
Triglycerides	93	1.00	0.02	1.00	1.5	1.0	2.0	−3.5	6.5
TSH	98	1.06	−0.01	0.99	2.8	1.1	4.4	−13.3	18.8
fT4	98	0.96	0.48	0.84	−0.3	−1.8	1.3	−15.5	15.0
Troponin-T	86	1.00	0.00	0.88	0.0	0	0	0	0
CK-MB	86	1.00	−0.03	1.00	−1.7	−2.5	−1.0	−8.6	5.1
NT-proBNP	85	1.01	−0.16	1.00	0.9	−0.7	2.4	−13.3	15.0
PSA	87	0.98	0.00	0.97	−2.1	−4.9	0.8	−28.3	24.1

Passing Bablok: slope, constant.

Correl: Spearman's correlation coefficient: rs

mBias%: Bland-Altman mean Bias with 95% confidence interval and 95% limits of agreement.

**Table 4:** Stability of serum in RSTs over 4 days indicated by repeat testing.

Analyte	Units	Range*	D1** *n* = 31	D2** *n* = 31	D3** *n* = 27	D4** *n* = 18	ANOVA
Urea	mmol/L	2.3, 75	4.24 (0.21)	4.17 (0.20)	4.13 (0.19)	4.07 (0.22)	NS
Creatinine	*μ*mol/L	47, 108	72.0 (2.80)	75.2 (2.91)	76.7 (3.03)	75.5 (2.88)	NS
Bicarbonate	mmol/L	22.0, 28.5	25.3 (0.31)	18.8 (0.39)	16.9 (0.25)	16.9 (0.30)	d2, d3, d4
Glucose	mmol/L	3.5, 9.0	4.97 (0.19)	5.06 (0.19)	5.16 (0.22)	5.21 (0.31)	NS
Sodium	mmol/L	135, 144	140 (0.40)	143 (0.49)	147 (0.53)	148 (0.78)	d3, d4
Potassium	mmol/L	3.4, 5.0	4.1 (0.05)	4.2 (0.05)	4.4 (0.07)	4.4 (0.08)	d3, d4
Chloride	mmol/L	101, 107	104 (0.32)	105 (0.40)	107 (0.37)	109 (0.51)	d3, d4
Total Protein	g/L	60, 80	71.0 (0.74)	72.2 (0.74)	73.6 (0.97)	73.4 (1.14)	NS
Albumin	g/L	34, 44	38.8 (0.45)	39.1 (0.49)	40.7 (0.53)	41.4 (0.63)	d3, d4
Total Bilirubin	*µ*mol/L	4.4, 23.5	9.69 (0.89)	8.95 (0.81)	8.79 (0.79)	8.26 (1.05)	NS
ALP	U/L	39, 331	73.4 (10.7)	75.9 (11.3)	81.8 (12.9)	95.8 (19.6)	NS
ALT	U/L	7, 36	16.4 (1.22)	16.1 (1.12)	15.3 (1.24)	16.0 (1.60)	NS
AST	U/L	10, 26	15.6 (0.71)	16.2 (0.70)	16.7 (0.85)	18.1 (1.11)	NS
Cholesterol	mmol/L	3.17, 6.27	4.84 (0.14)	4.91 (0.14)	4.99 (0.16)	4.89 (0.20)	NS
HDL	mmol/L	0.79, 2.75	1.47 (0.06)	1.46 (0.06)	1.51 (0.07)	1.52 (0.10)	NS
Triglycerides	mmol/L	0.42, 2.88	1.18 (0.10)	1.22 (0.10)	1.26 (0.11)	1.33 (0.16)	NS
TSH	mU/L	0.487, 6.20	1.889 (0.21)	1.893 (0.21)	2.010 (0.24)	2.360 (0.34)	NS
fT4	pmol/L	8.7, 14.6	11.3 (0.24)	10.9 (0.24)	11.1 (0.26)	11.2 (0.36)	NS
CK-MB	*μ*g/L	0.88, 4.82	1.80 (0.15)	1.88 (0.14)	1.65 (0.14)	1.61 (0.21)	NS
NT-proBNP	pg/mL	5.0, 100.6	35.9 (4.98)	37.3 (4.97)	37.9 (5.28)	47.5 (7.00)	NS

*Range: minimum, maximum.

**D1: day 1, D2: day 2, D3: day 3, D4: day 4.

Result: mean (SD).

d2, d3, d4: *P* < 0.05.

Note: same stability shown on SST tubes.
